# Supplementary material for: Power shift in the transformation and upgrading of the service sector—Empirical evidence from China
Source: PLoS One. 2025 Apr 23;20(4):e0317800. doi: 10.1371/journal.pone.0317800 (PMC12017479; doi:10.1371/journal.pone.0317800)
Supplement: S1 File — . (DOCX) [file pone.0317800.s001.docx]

# Appendix A

Appendix A gives the green technology efficiency of each province and the composition of the production frontier in the corresponding years, using the input and output data in the development of the service sector in 30 provinces in mainland China from 2003 to 2019 as a sample.

**Table A.1** Green Technology Efficiency in Service Industries of China’s Provinces

| **Province** | **2003** | **2004** | **2005** | **2006** | **2007** | **2008** | **2009** | **2010** | **2011** |
| --- | --- | --- | --- | --- | --- | --- | --- | --- | --- |
| Beijing | 1.0000 | 1.0000 | 1.0000 | 1.0000 | 1.0000 | 1.0000 | 1.0000 | 1.0000 | 1.0000 |
| Tianjin | 1.0000 | 0.7825 | 0.7803 | 0.7842 | 0.7861 | 0.7803 | 0.7616 | 0.7738 | 0.7781 |
| Hebei | 0.6807 | 0.6821 | 0.6958 | 0.6932 | 0.6961 | 0.7135 | 0.7024 | 0.6982 | 0.6641 |
| Shanxi | 0.6148 | 0.6223 | 0.6313 | 0.6197 | 0.6242 | 0.6258 | 0.6178 | 0.6097 | 0.5760 |
| Inner Mongoria IM | 0.6278 | 0.6410 | 0.6583 | 0.6571 | 0.6689 | 0.6602 | 0.6503 | 0.6395 | 0.5527 |
| Liaoning | 0.6632 | 0.6670 | 0.6612 | 0.6629 | 0.6594 | 0.6707 | 0.6684 | 0.6622 | 0.6320 |
| Jilin | 0.6292 | 0.6350 | 0.6426 | 0.6502 | 0.6603 | 0.6710 | 0.6821 | 0.6759 | 0.6396 |
| Heilongjiang | 0.6748 | 0.6669 | 0.6702 | 0.6724 | 0.6744 | 0.6791 | 0.6838 | 0.6671 | 0.6164 |
| Shanghai | 1.0000 | 1.0000 | 1.0000 | 1.0000 | 1.0000 | 1.0000 | 1.0000 | 1.0000 | 1.0000 |
| Jiangsu | 0.7531 | 0.7537 | 0.7647 | 0.7794 | 0.8240 | 1.0000 | 1.0000 | 1.0000 | 1.0000 |
| Zhejiang | 0.8468 | 1.0000 | 1.0000 | 1.0000 | 1.0000 | 1.0000 | 1.0000 | 1.0000 | 1.0000 |
| Anhui | 0.6708 | 0.6817 | 0.6833 | 0.6714 | 0.6660 | 0.6657 | 0.6633 | 0.6525 | 0.6246 |
| Fujian | 1.0000 | 1.0000 | 1.0000 | 1.0000 | 1.0000 | 1.0000 | 1.0000 | 0.9118 | 0.7749 |
| Jiangxi | 0.6631 | 0.6515 | 0.6631 | 0.6598 | 0.6558 | 0.6646 | 0.6693 | 0.6640 | 0.6777 |
| Shandong | 0.7011 | 0.7063 | 0.7201 | 0.7284 | 1.0000 | 1.0000 | 0.7871 | 0.7416 | 0.6586 |
| Henan | 1.0000 | 1.0000 | 0.6992 | 0.6917 | 0.6908 | 0.6948 | 0.6940 | 0.6813 | 0.6394 |
| Hubei | 0.6553 | 0.6645 | 0.6805 | 0.6780 | 0.6920 | 0.7226 | 0.7042 | 0.6904 | 0.6747 |
| Hunan | 1.0000 | 1.0000 | 0.6681 | 0.6616 | 1.0000 | 0.7117 | 0.6779 | 0.6748 | 0.6838 |
| Guangdong | 1.0000 | 1.0000 | 1.0000 | 1.0000 | 1.0000 | 1.0000 | 1.0000 | 1.0000 | 1.0000 |
| Guangxi | 0.6612 | 0.6490 | 0.6470 | 0.6472 | 0.6601 | 0.6707 | 0.6924 | 0.6818 | 0.6934 |
| Hainan | 1.0000 | 1.0000 | 1.0000 | 1.0000 | 1.0000 | 1.0000 | 1.0000 | 1.0000 | 0.7969 |
| Chongqing | 0.6811 | 0.6813 | 0.6841 | 0.6919 | 0.6950 | 0.7003 | 0.6981 | 0.6911 | 0.6699 |
| Sichuan | 0.6500 | 0.6537 | 0.6795 | 0.6732 | 0.6831 | 0.6814 | 0.6798 | 0.6844 | 0.6891 |
| Guizhou | 0.6385 | 0.6348 | 0.6488 | 0.6449 | 0.6761 | 0.6628 | 0.6527 | 0.6470 | 0.6235 |
| Yunnan | 0.7102 | 0.6969 | 0.7120 | 0.6960 | 0.6907 | 0.6903 | 0.6909 | 0.6826 | 0.6479 |
| Shaanxi | 0.6514 | 0.6341 | 0.6369 | 0.6331 | 0.6369 | 0.6384 | 0.6615 | 0.6555 | 0.6015 |
| Gansu | 0.6427 | 0.6489 | 0.6776 | 0.6792 | 0.6758 | 0.6795 | 0.6786 | 0.6682 | 0.6076 |
| Qinghai | 0.6920 | 0.6735 | 0.6378 | 0.6333 | 0.6258 | 0.6357 | 0.6416 | 0.6343 | 0.5966 |
| Ningxia | 0.5438 | 0.5591 | 0.5319 | 0.5210 | 0.5242 | 0.5392 | 0.5310 | 0.5235 | 0.4914 |
| Xinjiang | 0.6726 | 0.6587 | 0.6691 | 0.6608 | 0.6640 | 0.6633 | 0.6578 | 0.6498 | 0.6185 |
| Mean | 0.7575 | 0.7548 | 0.7381 | 0.7363 | 0.7610 | 0.7607 | 0.7516 | 0.7420 | 0.7076 |

**Table A.2** Cont’d table

| **Province** | **2012** | **2013** | **2014** | **2015** | **2016** | **2017** | **2018** | **2019** |
| --- | --- | --- | --- | --- | --- | --- | --- | --- |
| Beijing | 1.0000 | 1.0000 | 1.0000 | 1.0000 | 1.0000 | 1.0000 | 1.0000 | 1.0000 |
| Tianjin | 0.7680 | 0.7704 | 0.7344 | 0.7232 | 0.7121 | 0.6904 | 0.6836 | 0.6914 |
| Hebei | 0.6553 | 0.6481 | 0.6452 | 0.6236 | 0.6399 | 0.6259 | 0.6304 | 0.6441 |
| Shanxi | 0.5612 | 0.5483 | 0.5395 | 0.5130 | 0.4973 | 0.5005 | 0.4988 | 0.5015 |
| Inner Mongoria IM | 0.5471 | 0.5335 | 0.5442 | 0.5351 | 0.5553 | 0.5773 | 0.5923 | 0.5936 |
| Liaoning | 0.6218 | 0.6244 | 0.6152 | 0.6216 | 0.6455 | 0.6304 | 0.6310 | 0.6255 |
| Jilin | 0.6349 | 0.6368 | 0.6267 | 0.6245 | 0.6356 | 0.6210 | 0.6069 | 0.6073 |
| Heilongjiang | 0.6020 | 0.5976 | 0.6065 | 0.6074 | 0.6049 | 0.6084 | 0.5994 | 0.5989 |
| Shanghai | 1.0000 | 1.0000 | 1.0000 | 1.0000 | 1.0000 | 1.0000 | 1.0000 | 1.0000 |
| Jiangsu | 1.0000 | 1.0000 | 0.8982 | 0.8403 | 0.7105 | 1.0000 | 0.6820 | 1.0000 |
| Zhejiang | 1.0000 | 1.0000 | 1.0000 | 1.0000 | 0.7756 | 0.7380 | 0.7410 | 0.7613 |
| Anhui | 0.6114 | 0.6039 | 0.5967 | 0.5892 | 0.5310 | 0.5233 | 0.5151 | 0.5386 |
| Fujian | 0.7607 | 0.7714 | 0.7384 | 0.7278 | 0.6909 | 0.6689 | 0.6524 | 0.6486 |
| Jiangxi | 0.6720 | 0.6708 | 0.6567 | 0.6667 | 0.5856 | 0.5782 | 0.5655 | 0.5733 |
| Shandong | 0.6544 | 0.6665 | 0.6591 | 0.6432 | 0.6588 | 0.6504 | 0.6588 | 0.6705 |
| Henan | 0.6387 | 0.6439 | 0.6457 | 0.6225 | 0.6345 | 0.6164 | 0.6098 | 0.6331 |
| Hubei | 0.6720 | 0.6840 | 0.6785 | 0.6824 | 0.6578 | 0.6382 | 0.6407 | 0.6407 |
| Hunan | 0.6851 | 0.6930 | 0.6899 | 0.6945 | 0.6649 | 0.6472 | 0.6444 | 0.6490 |
| Guangdong | 1.0000 | 1.0000 | 1.0000 | 1.0000 | 1.0000 | 0.8024 | 0.7162 | 0.6896 |
| Guangxi | 0.6926 | 0.6956 | 0.6883 | 0.6861 | 0.7595 | 0.6577 | 0.7010 | 1.0000 |
| Hainan | 1.0000 | 1.0000 | 1.0000 | 1.0000 | 0.6808 | 0.6279 | 0.6090 | 0.6395 |
| Chongqing | 0.6712 | 0.6918 | 0.6942 | 0.7015 | 0.7193 | 0.7044 | 0.7107 | 0.7065 |
| Sichuan | 0.6851 | 0.6855 | 0.6798 | 0.6950 | 0.6891 | 0.6862 | 0.6671 | 0.6534 |
| Guizhou | 0.6057 | 0.5968 | 0.6292 | 0.6174 | 0.6014 | 0.5931 | 0.5881 | 0.5887 |
| Yunnan | 0.6317 | 0.6400 | 0.6324 | 0.6416 | 0.6094 | 0.6035 | 0.5803 | 0.5809 |
| Shaanxi | 0.6576 | 0.6616 | 0.6607 | 0.6206 | 0.6264 | 0.6057 | 0.5880 | 0.5601 |
| Gansu | 0.6000 | 0.5961 | 0.5961 | 0.5827 | 0.5879 | 0.5779 | 0.5676 | 0.5760 |
| Qinghai | 0.5698 | 0.5531 | 0.5643 | 0.5692 | 0.5575 | 0.5676 | 0.5440 | 0.5518 |
| Ningxia | 0.4759 | 0.4588 | 0.4705 | 0.4493 | 0.4324 | 0.4307 | 0.4231 | 0.4242 |
| Xinjiang | 0.6106 | 0.6007 | 0.5911 | 0.5730 | 0.5511 | 0.5359 | 0.5187 | 0.5176 |
| Mean | 0.7095 | 0.7091 | 0.7027 | 0.6950 | 0.6672 | 0.6569 | 0.6389 | 0.6622 |

Appendix B

**Table B.1** Green Technology Efficiency in Service Industries of China’s Provinces:2003-2019

| **Province** | **2003-2004** | | | | |
| --- | --- | --- | --- | --- | --- |
|  | **GTFP** | **Green pure te** | **Green-scale efficiency** | **te** | **tc** |
| Beijing | 0.9740 | 1.0000 | 1.0000 | 1.0000 | 0.9740 |
| Tianjin | 0.9786 | 1.0000 | 0.7825 | 0.7825 | 1.2505 |
| Hebei | 0.9849 | 1.0019 | 1.0002 | 1.0021 | 0.9829 |
| Shanxi | 1.0032 | 1.6093 | 0.6291 | 1.0123 | 0.9910 |
| Inner Mongoria IM | 0.9983 | 1.0315 | 0.9898 | 1.0210 | 0.9777 |
| Liaoning | 0.9839 | 1.0113 | 0.9946 | 1.0058 | 0.9782 |
| Jilin | 0.9953 | 1.0176 | 0.9918 | 1.0093 | 0.9861 |
| Heilongjiang | 0.9819 | 0.9923 | 0.9959 | 0.9882 | 0.9936 |
| Shanghai | 0.9983 | 1.0000 | 1.0000 | 1.0000 | 0.9983 |
| Jiangsu | 0.9670 | 1.0000 | 1.0008 | 1.0008 | 0.9663 |
| Zhejiang | 0.9971 | 1.1234 | 1.0512 | 1.1810 | 0.8443 |
| Anhui | 0.9818 | 1.0197 | 0.9967 | 1.0163 | 0.9660 |
| Fujian | 0.8649 | 1.0000 | 1.0000 | 1.0000 | 0.8649 |
| Jiangxi | 0.9813 | 0.9869 | 0.9955 | 0.9825 | 0.9988 |
| Shandong | 0.9830 | 1.0000 | 1.0074 | 1.0074 | 0.9757 |
| Henan | 0.6776 | 1.0000 | 1.0000 | 1.0000 | 0.6776 |
| Hubei | 0.9838 | 1.0149 | 0.9991 | 1.0140 | 0.9703 |
| Hunan | 0.6627 | 1.0000 | 1.0000 | 1.0000 | 0.6627 |
| Guangdong | 0.9249 | 1.0000 | 1.0000 | 1.0000 | 0.9249 |
| Guangxi | 0.9626 | 0.9810 | 1.0005 | 0.9815 | 0.9807 |
| Hainan | 0.8819 | 1.0000 | 1.0000 | 1.0000 | 0.8819 |
| Chongqing | 0.9859 | 1.0073 | 0.9932 | 1.0004 | 0.9855 |
| Sichuan | 0.9837 | 1.0079 | 0.9979 | 1.0057 | 0.9781 |
| Guizhou | 0.9862 | 1.0026 | 0.9917 | 0.9943 | 0.9919 |
| Yunnan | 0.9662 | 0.9953 | 0.9858 | 0.9812 | 0.9847 |
| Shaanxi | 0.9765 | 0.9784 | 0.9948 | 0.9733 | 1.0033 |
| Gansu | 0.9931 | 1.0330 | 0.9774 | 1.0097 | 0.9836 |
| Qinghai | 0.9944 | 1.0000 | 0.9733 | 0.9733 | 1.0217 |
| Ningxia | 0.9851 | 0.6827 | 1.5059 | 1.0280 | 0.9582 |
| Xinjiang | 0.9937 | 0.9910 | 0.9882 | 0.9794 | 1.0147 |
| Mean | 0.9544 | 1.0163 | 0.9948 | 0.9983 | 0.9589 |

**Table B.2** Cont’d table

| **Province** | **2011-2012** | | | | |
| --- | --- | --- | --- | --- | --- |
|  | **GTFP** | **Green pure te** | **Green-scale efficiency** | **te** | **tc** |
| Beijing | 1.0031 | 1.0000 | 1.0000 | 1.0000 | 1.0031 |
| Tianjin | 0.9954 | 1.0005 | 0.9866 | 0.9871 | 1.0084 |
| Hebei | 0.9850 | 0.9871 | 0.9996 | 0.9867 | 0.9982 |
| Shanxi | 0.9747 | 0.9750 | 0.9994 | 0.9744 | 1.0003 |
| Inner Mongoria IM | 0.9865 | 0.9909 | 0.9990 | 0.9899 | 0.9966 |
| Liaoning | 0.9810 | 0.9829 | 1.0009 | 0.9838 | 0.9971 |
| Jilin | 0.9932 | 0.9935 | 0.9991 | 0.9926 | 1.0006 |
| Heilongjiang | 0.9871 | 0.9780 | 0.9986 | 0.9766 | 1.0107 |
| Shanghai | 1.0214 | 1.0000 | 1.0000 | 1.0000 | 1.0214 |
| Jiangsu | 1.0008 | 1.0000 | 1.0000 | 1.0000 | 1.0008 |
| Zhejiang | 1.0131 | 1.0000 | 1.0000 | 1.0000 | 1.0131 |
| Anhui | 0.9803 | 0.9698 | 1.0092 | 0.9788 | 1.0016 |
| Fujian | 0.9967 | 0.9833 | 0.9984 | 0.9818 | 1.0153 |
| Jiangxi | 0.9968 | 0.9937 | 0.9979 | 0.9916 | 1.0053 |
| Shandong | 0.9897 | 0.9911 | 1.0026 | 0.9937 | 0.9960 |
| Henan | 0.9950 | 1.0034 | 0.9956 | 0.9990 | 0.9960 |
| Hubei | 0.9941 | 0.9958 | 1.0001 | 0.9960 | 0.9981 |
| Hunan | 1.0032 | 1.0039 | 0.9980 | 1.0019 | 1.0013 |
| Guangdong | 1.0104 | 1.0000 | 1.0000 | 1.0000 | 1.0104 |
| Guangxi | 0.9955 | 1.0070 | 0.9919 | 0.9989 | 0.9966 |
| Hainan | 0.9814 | 1.0000 | 1.2549 | 1.2549 | 0.7821 |
| Chongqing | 1.0032 | 1.0060 | 0.9959 | 1.0019 | 1.0013 |
| Sichuan | 0.9927 | 1.0001 | 0.9942 | 0.9943 | 0.9985 |
| Guizhou | 0.9681 | 0.9745 | 0.9970 | 0.9715 | 0.9964 |
| Yunnan | 0.9797 | 0.9722 | 1.0029 | 0.9750 | 1.0049 |
| Shaanxi | 1.0653 | 1.1286 | 0.9686 | 1.0932 | 0.9744 |
| Gansu | 0.9905 | 1.0047 | 0.9829 | 0.9874 | 1.0031 |
| Qinghai | 0.9673 | 1.0000 | 0.9551 | 0.9551 | 1.0128 |
| Ningxia | 0.9724 | 1.4328 | 0.6759 | 0.9684 | 1.0041 |
| Xinjiang | 0.9939 | 1.0000 | 0.9874 | 0.9874 | 1.0066 |
| Mean | 0.9939 | 1.0125 | 0.9931 | 1.0007 | 0.9952 |

**Table B.3** Cont’d table

| **Province** | **2018-2019** | | | | |
| --- | --- | --- | --- | --- | --- |
|  | **GTFP** | **Green pure te** | **Green-scale efficiency** | **te** | **tc** |
| Beijing | 1.1239 | 1.0000 | 1.0000 | 1.0000 | 1.1239 |
| Tianjin | 1.0189 | 1.0132 | 0.9983 | 1.0115 | 1.0074 |
| Hebei | 1.0293 | 1.0147 | 1.0070 | 1.0218 | 1.0073 |
| Shanxi | 1.0196 | 1.0113 | 0.9940 | 1.0053 | 1.0143 |
| Inner Mongoria IM | 1.0121 | 1.0013 | 1.0008 | 1.0021 | 1.0100 |
| Liaoning | 0.9998 | 0.9934 | 0.9979 | 0.9913 | 1.0086 |
| Jilin | 1.0069 | 0.9986 | 1.0020 | 1.0006 | 1.0063 |
| Heilongjiang | 1.0063 | 0.9954 | 1.0038 | 0.9992 | 1.0071 |
| Shanghai | 1.0000 | 1.0000 | 1.0000 | 1.0000 | 1.0000 |
| Jiangsu | 1.4681 | 1.0000 | 1.4663 | 1.4663 | 1.0013 |
| Zhejiang | 1.0327 | 1.0000 | 1.0273 | 1.0273 | 1.0053 |
| Anhui | 1.0461 | 1.0202 | 1.0250 | 1.0458 | 1.0003 |
| Fujian | 0.9949 | 0.9937 | 1.0004 | 0.9941 | 1.0009 |
| Jiangxi | 1.0158 | 1.0146 | 0.9993 | 1.0139 | 1.0019 |
| Shandong | 1.0268 | 1.0000 | 1.0178 | 1.0178 | 1.0088 |
| Henan | 1.0440 | 1.0162 | 1.0217 | 1.0382 | 1.0055 |
| Hubei | 1.0154 | 1.0003 | 0.9998 | 1.0001 | 1.0152 |
| Hunan | 1.0166 | 1.0050 | 1.0020 | 1.0070 | 1.0095 |
| Guangdong | 0.9640 | 1.0000 | 0.9628 | 0.9628 | 1.0012 |
| Guangxi | 1.4265 | 1.0000 | 1.4265 | 1.4265 | 1.0000 |
| Hainan | 1.0501 | 1.0000 | 1.0501 | 1.0501 | 1.0000 |
| Chongqing | 1.0161 | 0.9668 | 1.0282 | 0.9940 | 1.0223 |
| Sichuan | 1.0087 | 0.9856 | 0.9938 | 0.9794 | 1.0299 |
| Guizhou | 1.0056 | 0.9948 | 1.0062 | 1.0009 | 1.0047 |
| Yunnan | 1.0083 | 1.0015 | 0.9996 | 1.0011 | 1.0072 |
| Shaanxi | 0.9610 | 0.9396 | 1.0139 | 0.9526 | 1.0088 |
| Gansu | 1.0212 | 1.0104 | 1.0044 | 1.0149 | 1.0062 |
| Qinghai | 1.0177 | 1.0000 | 1.0144 | 1.0144 | 1.0032 |
| Ningxia | 1.0019 | 0.6269 | 1.5991 | 1.0025 | 0.9994 |
| Xinjiang | 1.0023 | 0.9666 | 1.0322 | 0.9977 | 1.0046 |
| Mean | 1.0454 | 0.9857 | 1.0565 | 1.0346 | 1.0107 |

Appendix C

**Table C.1** Sources of GTFP Growth in China's Provincial Services: cumulative decomposition

| **Province** | **GTFP** | **Green pure te** | **Green-scale efficiency** | **te** | **tc** |
| --- | --- | --- | --- | --- | --- |
| Beijing | 1.6280 | 1.6280 | 1.0000 | 1.0000 | 1.0000 |
| Tianjin | 1.1142 | 1.6116 | 0.6914 | 0.7782 | 0.8884 |
| Hebei | 0.9693 | 1.0243 | 0.9463 | 0.9517 | 0.9943 |
| Shanxi | 0.8214 | 1.0070 | 0.8157 | 0.8436 | 0.9670 |
| Inner Mongoria IM | 1.0254 | 1.0845 | 0.9455 | 0.9756 | 0.9691 |
| Liaoning | 1.0229 | 1.0845 | 0.9432 | 0.9458 | 0.9972 |
| Jilin | 1.0266 | 1.0635 | 0.9653 | 1.0104 | 0.9554 |
| Heilongjiang | 0.9779 | 1.1019 | 0.8875 | 0.8920 | 0.9950 |
| Shanghai | 1.5493 | 1.5493 | 1.0000 | 1.0000 | 1.0000 |
| Jiangsu | 1.4223 | 1.0711 | 1.3279 | 1.0000 | 1.3279 |
| Zhejiang | 1.2235 | 1.3609 | 0.8991 | 1.1234 | 0.8003 |
| Anhui | 0.8087 | 1.0071 | 0.8030 | 0.8477 | 0.9472 |
| Fujian | 0.6486 | 1.0000 | 0.6486 | 0.6492 | 0.9990 |
| Jiangxi | 0.9544 | 1.1039 | 0.8646 | 0.8724 | 0.9911 |
| Shandong | 0.9938 | 1.0391 | 0.9564 | 1.0000 | 0.9564 |
| Henan | 0.6331 | 1.0000 | 0.6331 | 0.6450 | 0.9815 |
| Hubei | 0.9784 | 1.0007 | 0.9777 | 0.9853 | 0.9923 |
| Hunan | 0.6490 | 1.0000 | 0.6490 | 0.6537 | 0.9928 |
| Guangdong | 0.8470 | 1.2282 | 0.6896 | 1.0000 | 0.6896 |
| Guangxi | 1.5372 | 1.0164 | 1.5123 | 1.5058 | 1.0043 |
| Hainan | 0.8490 | 1.3277 | 0.6395 | 1.0000 | 0.6395 |
| Chongqing | 1.1714 | 1.1293 | 1.0373 | 1.1294 | 0.9184 |
| Sichuan | 1.0651 | 1.0595 | 1.0053 | 1.0112 | 0.9942 |
| Guizhou | 1.0124 | 1.0980 | 0.9220 | 0.9591 | 0.9613 |
| Yunnan | 0.9302 | 1.1373 | 0.8179 | 0.8156 | 1.0028 |
| Shaanxi | 0.9464 | 1.1006 | 0.8598 | 0.8705 | 0.9878 |
| Gansu | 0.9768 | 1.0898 | 0.8963 | 0.9653 | 0.9285 |
| Qinghai | 0.9387 | 1.1771 | 0.7974 | 1.0000 | 0.7974 |
| Ningxia | 0.8056 | 1.0329 | 0.7800 | 0.6269 | 1.2442 |
| Xinjiang | 0.8819 | 1.1460 | 0.7695 | 0.7806 | 0.9858 |
| Mean | 1.0136 | 1.1427 | 0.8894 | 0.9279 | 0.9636 |

Appendix D

**Table D.1** Sources of GTFP Growth in China's Provincial Services: cumulative decomposition (%)

| **Province** | **Labor productivity** | **GTFP** | **Green pure te** | **Green-scale efficiency** | **te** | **tc** | **Input element** |
| --- | --- | --- | --- | --- | --- | --- | --- |
| Beijing | 1.68 | 93.67 | 0.00 | 0.00 | 0.00 | 93.67 | 6.33 |
| Tianjin | 2.03 | 15.32 | -35.51 | -16.76 | -52.26 | 67.58 | 84.68 |
| Hebei | 3.18 | -2.70 | -4.28 | -0.49 | -4.78 | 2.08 | 102.70 |
| Shanxi | 2.30 | -23.67 | -20.48 | -4.04 | -24.52 | 0.84 | 123.67 |
| Inner Mongoria IM | 3.05 | 2.25 | -2.22 | -2.81 | -5.03 | 7.28 | 97.75 |
| Liaoning | 2.63 | 2.34 | -5.75 | -0.29 | -6.04 | 8.38 | 97.66 |
| Jilin | 2.91 | 2.46 | 0.97 | -4.27 | -3.31 | 5.77 | 97.54 |
| Heilongjiang | 2.49 | -2.45 | -12.51 | -0.55 | -13.06 | 10.61 | 102.45 |
| Shanghai | 2.04 | 61.46 | 0.00 | 0.00 | 0.00 | 61.46 | 38.54 |
| Jiangsu | 3.74 | 26.68 | 0.00 | 21.48 | 21.48 | 5.20 | 73.32 |
| Zhejiang | 2.83 | 19.40 | 11.20 | -21.43 | -10.23 | 29.64 | 80.60 |
| Anhui | 2.73 | -21.16 | -16.46 | -5.40 | -21.86 | 0.70 | 121.16 |
| Fujian | 2.06 | -59.82 | -59.68 | -0.14 | -59.82 | 0.00 | 159.82 |
| Jiangxi | 3.07 | -4.16 | -12.17 | -0.80 | -12.96 | 8.81 | 104.16 |
| Shandong | 3.69 | -0.48 | 0.00 | -3.41 | -3.41 | 2.94 | 100.48 |
| Henan | 2.42 | -51.65 | -49.54 | -2.11 | -51.65 | 0.00 | 151.65 |
| Hubei | 3.94 | -1.59 | -1.08 | -0.57 | -1.64 | 0.05 | 101.59 |
| Hunan | 3.64 | -33.47 | -32.91 | -0.56 | -33.47 | 0.00 | 133.47 |
| Guangdong | 1.70 | -31.14 | 0.00 | -69.68 | -69.68 | 38.54 | 131.14 |
| Guangxi | 3.86 | 31.82 | 30.29 | 0.32 | 30.61 | 1.21 | 68.18 |
| Hainan | 2.13 | -21.62 | 0.00 | -59.06 | -59.06 | 37.44 | 121.62 |
| Chongqing | 3.20 | 13.61 | 10.47 | -7.32 | 3.15 | 10.46 | 86.39 |
| Sichuan | 3.42 | 5.13 | 0.90 | -0.47 | 0.43 | 4.70 | 94.87 |
| Guizhou | 3.78 | 0.92 | -3.14 | -2.97 | -6.11 | 7.03 | 99.08 |
| Yunnan | 1.86 | -11.63 | -32.75 | 0.45 | -32.29 | 20.67 | 111.63 |
| Shaanxi | 3.00 | -5.02 | -12.63 | -1.12 | -13.76 | 8.74 | 105.02 |
| Gansu | 3.35 | -1.94 | -2.92 | -6.13 | -9.05 | 7.11 | 101.94 |
| Qinghai | 2.36 | -7.37 | 0.00 | -26.37 | -26.37 | 18.99 | 107.37 |
| Ningxia | 1.74 | -38.94 | -84.14 | 39.37 | -44.77 | 5.83 | 138.94 |
| Xinjiang | 1.79 | -21.67 | -42.68 | -2.46 | -45.14 | 23.48 | 121.67 |
| Mean | 2.75 | -2.18 | -12.57 | -5.92 | -18.49 | 16.31 | 102.18 |
