# Supplementary material for: Power shift in the transformation and upgrading of the service sector—Empirical evidence from China
Source: PLoS One. 2025 Apr 23;20(4):e0317800. doi: 10.1371/journal.pone.0317800 (PMC12017479; doi:10.1371/journal.pone.0317800)
Supplement: S1 Text — . (DOCX) [file pone.0317800.s002.docx]

**Figure 1.** Factors influencing GTFP in the service sector.

GTC

GTFP growth

GEC

Internal composition

Energy structure

Environmental regulation

Factor endowment

Economic development level

Services development level

Degree of informatization

Government control
