# Supplementary material for: Power shift in the transformation and upgrading of the service sector—Empirical evidence from China
Source: PLoS One. 2025 Apr 23;20(4):e0317800. doi: 10.1371/journal.pone.0317800 (PMC12017479; doi:10.1371/journal.pone.0317800)
Supplement: Attachment — Submitted filename: Response to Reviewers.docx [file pone.0317800.s004.docx]

**Response to Reviewers**

**Dear Reviewer:**

We would like to express our heartfelt gratitude to two anonymous reviewers for their valuable feedback. Our manuscript, titled “**Power shift in the transformation and upgrading of the service sector --- Empirical evidence from China**”, benefited significantly from the constructive comments and insights from the review team. Based on the suggestions received, we have made careful revisions to the original manuscript. In the revised manuscript, all changes are marked in red. In addition, we have also carefully proofread this manuscript for typographical, grammatical, and other errors. We hope the revised manuscript is able to meet your standard of quality and address the concerns raised by the reviewers. In the following, you will find our detailed point-by-point responses to the reviewers’ comments.

**Reviewer #1**

We sincerely appreciate your valuable feedback on our manuscript. All of these comments have helped us significantly in improving the quality of our manuscript. Based on your comments and suggestions, we have made extensive changes to the manuscript in the revised manuscript. Our responses to your specific comments are provided below. We also provide a copy of the relevant sections (marked in red) following our responses.

**Major Comments**

(1) **Overall, this paper lacks a clear theoretical contribution. That is, what can we learn from the Chinese case? The authors need to point it out clearly in the parts of introduction and conclusion.**

***Response：***thanks for your valuable suggestions. Your suggestions made a huge improvement in our research paper. We have pointed it out clearly in the parts of introduction and conclusion. The revised text reads as follows:

**line 60**

...transformation to some extent. Based on China's experience, it has been found that the rapid growth of the service sector has come at the cost of high inputs, and that the relative contribution of green total factor productivity is much lower than that of input factor accumulation. GTFP in services is all affected by a variety of factors, most of which are closely related to the supply side. In the new era of...

**line 921**

Fourth, according to the empirical evidence from China, it has been found that the influencing factors of GTFP...

(2) **My first and primary concern lies in the novelty of this work, as I feel that the novelty issue has not been sufficiently highlighted in the current version. An important question shall be answered: does this work fill up some knowledge gaps which previous articles cannot address?**

***Response：***This is a very important revision proposal, and we fully agree with this suggestion and have made serious revisions. The revised text reads as follows:

**line 123**

...the existing literature. Our research fills a gap in this area. For this reason, this paper incorporates environmental constraints into the analysis of the transformation and upgrading of the service industry, analyzes it from the supply-side perspective, analyzes its future direction at the stage of high-quality development with the help of the green economic growth accounting model, and analyzes its influencing factors.

(3) **I suggest the authors to rewrite the abstract with a focus on background,objectives methodology, main findings and conclusion. Please add a sentence which shows the necessity of the study.**

***Response：***Thank you for pointing this out, we have rewritten the abstract. The revised text reads as follows:

**Line 21**

**Abstract:** In recent years, as the global economy has entered the "service economy" period, improving the service industry's total factor productivity, realizing high-quality development, and better playing its supportive role in the real economy and its function of improving people's well-being have become the top priorities for its development. The service industry is growing fast, yet its production and operations are putting pressure on the environment. For this reason, this paper incorporates environmental constraints into the analysis of the transformation and upgrading of the service industry to decompose and analyze the sources of green growth of the service industry. We find that: first, the level of green technology efficiency in the service industry is low, there is a downward trend, and the green technology efficiency gap widens significantly across provinces. Secondly, GTFP in the service sector is declining in general, with large inter-provincial differences, and the obstacle to growth lies in the deterioration of green technical efficiency, while the driving force for growth lies in green technological progress. Thirdly, labor productivity in the service sector has improved considerably, but all at the cost of high inputs, and the growth contribution of green total factor productivity and its constituent factors is negative, and the whole is still in the input factor-driven stage. Finally, the factors affecting GTFP in services are multifaceted, mainly involving structural, environmental regulatory, economic, social and institutional factors. This paper offers a theoretical foundation for further promoting the transformation and upgrading of the service industry to attract jade.

(4) **The"Conclusion" section should be largely improved, and the discussion and policy implication should be provided. The significance of the study should be enhanced in the Abstract,Introduction and Conclusion. The shortcoming of the study as well as more research and depth in the assessment and suggestions for the future should be presented.**

***Response：***Thank you for pointing this out, to address these issues, we have made changes, we have added a research significance to the abstract section, which mainly describes the conclusions reached in the study in China, in addition to the conclusions section.The revised text reads as follows:

**Line 38**

...factors. This paper offers a theoretical foundation for further promoting the transformation and upgrading of the service industry to attract jade.

**Line 72**

...pressure. To this end, this paper incorporates environmental constraints into the analysis of transformation and upgrading of the service industry, analyzes the transformation and upgrading of the service industry's power transformation history and future direction from the supply-side perspective with the help of the green economic growth accounting model, and analyzes the influencing factors of the transformation and upgrading of the innovation-driven service industry from the perspective of enhancing the green total factor productivity.

**Line 934**

This paper incorporates environmental constraints into the analysis of service industry transformation and upgrading, analyzes the power transformation process and future direction of service industry transformation and upgrading at the stage of high-quality development from the supply-side perspective with the help of green economic growth accounting and analysis model, and analyzes the influencing factors of transformation and upgrading of the innovation-driven service industry from the perspective of enhancing green total factor productivity....

(5) **Whether the table in the appendix can be put into the Manuscript？**

***Response：***Thank you for pointing this out. Due to the length of the article, some of the research data has been placed in an appendix.

**Minor comments:**

**(1) Add a separator for the numbers over 1.000. Check all numbers including those in the tables/figures.**

***Response：*** Thank you for pointing this out. We have carefully corrected them in our revised manuscript. The specific changes are as follows:

**Table 1.**

Statistical description of services input, output indicators: 2003-2019

| **variant** | **observed value** | **average value** | **upper quartile** | **standard deviation** | **minimum value** | **maximum values** |
| --- | --- | --- | --- | --- | --- | --- |
| gdp | 510 | 4,696.94 | 3,220.56 | 4,583.55 | 137.84 | 25,578.1 |
| k | 510 | 26,103.53 | 16,710.85 | 26,739.68 | 611.14 | 143,359.78 |
| H | 510 | 7,941.96 | 6,971.37 | 5,115.62 | 567.23 | 32,567.41 |
| S02 | 510 | 1.48 | 1.12 | 1.35 | 0.00 | 7.60 |
| COD | 510 | 9.76 | 7.16 | 8.36 | 0.48 | 42.84 |
| CO2 | 510 | 8,568.94 | 6,549.12 | 7,107.23 | 196.24 | 37,957.45 |

**(2) The fonts, paragraphs and table formatting need further beautification.**

***Response：***Thank you for pointing this out.We have further beautified the fonts, paragraphs and table formatting.

**(3) Remove the space after CO2 in line 302.**

***Response：***Thank you for pointing this out. We have carefully corrected them in our revised manuscript. The specific changes are as follows:

**line 302**

...variation of carbon dioxide emissions (CO2) has the smallest...

**(4) The font should be centered within the table.**

***Response：***Thank you for pointing this out. We sincerely apologize and have carefully corrected them in our revised manuscript. The specific changes are as follows:

**Table 1.**

Statistical description of services input, output indicators: 2003-2019

| **variant** | **observed value** | **average value** | **upper quartile** | **standard deviation** | **minimum value** | **maximum values** |
| --- | --- | --- | --- | --- | --- | --- |
| gdp | 510 | 4,696.94 | 3,220.56 | 4,583.55 | 137.84 | 25,578.1 |
| k | 510 | 26,103.53 | 16,710.85 | 26,739.68 | 611.14 | 143,359.78 |
| H | 510 | 7,941.96 | 6,971.37 | 5,115.62 | 567.23 | 32,567.41 |
| S02 | 510 | 1.48 | 1.12 | 1.35 | 0.00 | 7.60 |
| COD | 510 | 9.76 | 7.16 | 8.36 | 0.48 | 42.84 |
| CO2 | 510 | 8,568.94 | 6,549.12 | 7,107.23 | 196.24 | 37,957.45 |

**Table 2.** List of potential influences on GTFP in the service sector

| **form** | **variant** | **Measurement indicators** |
| --- | --- | --- |
| Structural factors | industry structure（*nbjg*） | Share of value added of three sectors, namely, accommodation and catering, wholesale and retail trade, and transportation, storage and postal services, in the value added of the service sector |
|  | Elementary structure（*ysjg*） | capital-labor ratio |
|  | energy structure（*nyjg*） | Share of industrial coal energy consumption in total industrial energy consumption |
| restraint | Administrative environmental regulation（*es*） | Investment in pollution control as a share of GDP |
|  | Market-based environmental regulation（*er*） | Revenue from sewage charges as a share of GDP |
| economic factor | Level of development of services（*savl*） | Value added of services as a share of GDP |
|  | Level of economic development（*pgdp*） | Natural logarithm of GDP per capita |
| social factor | degree of informatization（*ict*） | Internet penetration |
| Institutional factors | Government regulatory capacity（*govp*） | Fiscal expenditure as a share of GDP |

**Table 3.** Statistical Description of Factors Influencing GTFP in Service Industries

| **variant** | **observed value** | **average value** | **upper quartile** | **standard deviation** | **minimum value** | **maximum values** |
| --- | --- | --- | --- | --- | --- | --- |
| *nbjg* | 480 | 0.45 | 0.46 | 0.09 | 0.21 | 0.77 |
| *lnpgdp* | 480 | 10.09 | 10.10 | 0.67 | 8.29 | 11.86 |
| *es* | 480 | 0.02 | 0.01 | 0.05 | 0.00 | 0.41 |
| *ysjg* | 480 | 5.50 | 4.57 | 3.96 | 0.70 | 26.46 |
| *sval* | 480 | 0.44 | 0.41 | 0.10 | 0.27 | 0.84 |
| *nyjg* | 480 | 0.10 | 0.06 | 0.12 | 0.00 | 0.58 |
| *ict* | 480 | 0.36 | 0.39 | 0.20 | 0.03 | 0.78 |
| *govp* | 480 | 0.22 | 0.20 | 0.10 | 0.08 | 0.63 |
| *hc* | 8.76 | 1.02 | 8.71 | 6.38 | 12.78 | 8.76 |

**Table 7.** Robustness tests of GTFP Influencing Factors in the Service Sector

| **variable** | **（1）** | **（2）** | **（3）** | **（4）** | **（5）** | **（6）** |
| --- | --- | --- | --- | --- | --- | --- |
|  | **Green total factor productivity** | **Green technological advances** | **Green technology efficiency** | **Green total factor productivity** | **Green technological advances** | **Green technology efficiency** |
| *nbjg* | -0.061 | 0.224*** | -0.345*** | -0.114 | 0.116 | -0.294*** |
|  | (-0.85) | (3.18) | (-3.51) | (-1.51) | (1.54) | (-3.48) |
| *lnpgdp* | -1.462*** | -1.103*** | -0.314 | -1.264*** | -1.503*** | 0.169 |
|  | (-8.75) | (-6.75) | (-1.37) | (-6.54) | (-7.76) | (0.78) |
| *lnpgdp^2^* | 0.063*** | 0.059*** | -0.002 | 0.051*** | 0.068*** | -0.013 |
|  | (6.82) | (6.53) | (-0.13) | (4.80) | (6.35) | (-1.10) |
| *es* | 0.385** | 0.343* | 0.058 |  |  |  |
|  | (2.05) | (1.86) | (0.22) |  |  |  |
| *er* |  |  |  | 39.900*** | 11.582 | 33.251** |
|  |  |  |  | (2.75) | (0.80) | (2.05) |
| *ysjg* | 0.001 | -0.005** | 0.007** | 0.002 | -0.002 | 0.003 |
|  | (0.63) | (-2.42) | (2.32) | (0.70) | (-0.95) | (1.33) |
| *sval* | -0.275*** | -0.141 | -0.108 | -0.237** | -0.155* | -0.033 |
|  | (-3.12) | (-1.63) | (-0.89) | (-2.54) | (-1.66) | (-0.31) |
| *nyjg* | 0.066 | 0.065 | 0.011 | 0.049 | 0.136*** | -0.070 |
|  | (1.44) | (1.45) | (0.17) | (1.02) | (2.83) | (-1.30) |
| *ict* | -0.576*** | -0.372*** | -0.200 | -0.608*** | -0.620*** | -0.060 |
|  | (-5.72) | (-3.78) | (-1.45) | (-5.61) | (-5.71) | (-0.50) |
| *govp* | -0.460*** | 0.091 | -0.601*** | -0.415*** | 0.075 | -0.413*** |
|  | (-3.77) | (0.77) | (-3.61) | (-3.14) | (0.56) | (-2.80) |
| *hc* |  |  |  |  |  |  |
|  |  |  |  |  |  |  |
| *_cons* | 9.695*** | 6.153*** | 4.677*** | 8.852*** | 9.397*** | 0.821 |
|  | (11.17) | (7.24) | (3.94) | (9.05) | (9.59) | (0.75) |
| Year FE | Yes | Yes | Yes | Yes | Yes | Yes |
| Province FE | Yes | Yes | Yes | Yes | Yes | Yes |
| N | 480 | 480 | 480 | 480 | 480 | 480 |
| F | 17.4893 | 10.7067 | 4.4783 | 15.7665 | 15.7669 | 2.884 |
| R^2^ | 0.820 | 0.710 | 0.733 | 0.820 | 0.821 | 0.750 |

**line 875**

| **variant** | **（7）** | **（8）** | **（9）** |
| --- | --- | --- | --- |
|  | **Green total factor productivity** | **Green technological advances** | **Green technology efficiency** |
| nbjg | -0.046 | 0.211*** | -0.313*** |
|  | (-0.64) | (2.99) | (-3.19) |
| lnpgdp | -1.466*** | -1.099*** | -0.323 |
|  | (-8.81) | (-6.74) | (-1.43) |
| lnpgdp^2^ | 0.063*** | 0.058*** | -0.001 |
|  | (6.85) | (6.53) | (-0.11) |
| es | 0.379** | 0.349* | 0.044 |
|  | (2.02) | (1.90) | (0.17) |
| er |  |  |  |
|  |  |  |  |
| ysjg | 0.001 | -0.005** | 0.007** |
|  | (0.56) | (-2.37) | (2.23) |
| sval | -0.260*** | -0.154* | -0.075 |
|  | (-2.95) | (-1.78) | (-0.62) |
| nyjg | 0.065 | 0.066 | 0.009 |
|  | (1.43) | (1.47) | (0.14) |
| ict | -0.567*** | -0.381*** | -0.179 |
|  | (-5.63) | (-3.86) | (-1.31) |
| govp | -0.444*** | 0.078 | -0.567*** |
|  | (-3.65) | (0.65) | (-3.43) |
| hc | 0.033* | -0.028* | 0.073*** |
|  | (1.93) | (-1.67) | (3.10) |
| _cons | 9.415*** | 6.391*** | 4.066*** |
|  | (10.73) | (7.43) | (3.41) |
| Year FE | Yes | Yes | Yes |
| Province FE | Yes | Yes | Yes |
| N | 480 | 480 | 480 |
| F | 16.2134 | 9.9574 | 5.0746 |
| R^2^ | 0.822 | 0.712 | 0.739 |

**(5)From 2003-2019 should be From 2003 to 2019 in line 317.**

***Response：***Thank you for pointing this out. We sincerely apologize and have carefully corrected them in our revised manuscript. The specific changes are as follows:

**line 317**

“From 2003 to 2019, the green technology efficiency...”

**(6)Line 446: Remove " and".**

***Response：***Thank you for pointing this out. We have carefully corrected them in our revised manuscript. The specific changes are as follows:

**Line 446**

...an important role, they are both important ways to promote...

**Reviewer #2:**

We sincerely appreciate your valuable feedback on our manuscript. All of these comments have helped us significantly in improving the quality of our manuscript. Based on your comments and suggestions, we have made extensive changes to the manuscript in the revised manuscript. Our responses to your specific comments are provided below. We also provide a copy of the relevant sections (marked in red) following our responses.

**(1)This article has done a lot of work, but there are some errors in some details. For example, there is a statement ambiguity in line 312; In line 357, decrease should be replaced by increase; The last sentence of line 908 is logically confused.**

***Response：***Thank you for pointing this out. We sincerely apologize and have carefully corrected them in our revised manuscript. The specific changes are as follows:

**line 312**

First, the GTFP of the service industry has widespread in the development of services.

**line 357**

...variation increase from 0.0045 volatility to 0.0098...

**line 908**

basically, but basically all of them have a significant effect on some of these constituent factors.

**(2)From the perspective of the explanation of the content of the article, line 329 lacks the necessary explanation for the occurrence of this situation; Line 609, the article uses the capital-labor ratio to measure the input of factors, and how to reflect the input of technical factors; On line 829, if the regression is not significant, is it still meaningful to discuss?**

***Response：***Thank you for pointing this out. We sincerely apologize and have carefully corrected them in our revised manuscript. The specific changes are as follows:

**line 329**

Third, an analysis of the article's measurement data reveals that there is no...

**Line 609**

The article uses the capital-labor ratio to measure factor inputs and does not address inputs of technological factors; furthermore, this paper uses the share of capital stock and employment in the service sector in each province to measure the capital-labor ratio.

**line 829**

Energy consumption structure with both green technical efficiency and GTFP is not significant. However, the effect of energy consumption structure on green technological progress is significantly positive, indicating that energy consumption structure has a facilitating effect on green technological progress in the service industry. So it is still meaningful to discuss.

**(3)From the title of the paper, a large section and table in the later part of this paper discuss the influence of other variables on GTFP. The title focuses on the appropriateness of power shift, and the power shift in this paper is not clearly explained, and the specific decomposition process of development level and efficiency is not shown in detail, which needs further supplement and explanation.**

***Response：***Thank you for this fantastic and useful suggestion. In response to these questions I offer the following explanation:

From the supply side, both input factor accumulation and total factor productivity enhancement are the driving forces for the development of the service industry, and as the level of development of the service industry increases, the driving force will shift from being input factor-driven to being efficiency-driven and innovation-driven, i.e., total factor productivity enhancement.

In this study, the gross output value of the service industry is taken as the desired output, chemical oxygen demand (COD), domestic sulfur dioxide (SO2) and carbon dioxide (CO2) emissions in wastewater are taken as the non-desired outputs, and labor and physical capital are taken as the input factors.

**(4)Finally, after the conclusion of the analysis, the paper does not put forward specific and feasible suggestions, which needs to be supplemented and strengthened.**

***Response：***Respected reviewer thank you very much for your valuable suggestions. Your suggestions made a huge improvement in our research paper. We sincerely apologize and have modified the conclusions section and added the recommendations. The specific changes are as follows:

**line 871**

Conclusions and implications

**line 910**

Based on the above findings, this article proposes the following policy recommendations:

First, driving digital transformation in the service sector. Promote the construction of new infrastructure, strengthen the modularization of service elements and the professional division of labor, and improve the quality of services and the convenience of intelligent transformation and upgrading. Improvement of the digital service industry ecosystem and diversification of service modes and contents. Supply-side and demand-side joint efforts to promote industrial agglomeration from the original geospatial agglomeration mode to the network virtual agglomeration mode change.

Second, promoting value chain upgrading in the service sector. Introducing new technologies to refine and deepen the processing of traditional service industries. Fostering new service industries. Promoting competitive productive service industries. Industry-university-research cooperation and strengthening of talent cultivation. Optimize the modern service ecosystem.

Third, vigorously developing productive service industries. Enhancing the level of agglomeration of productive services and strengthening the degree of specialized agglomeration of productive services. Adopting information technology to respond to market demand in a timely manner. Select potential industries and enterprises to focus on breakthroughs.
